# Bouldering-Psychotherapy for treating depression in adolescents (BooSt): study protocol for a randomized controlled trial

**DOI:** 10.1186/s12887-026-07935-8

**Published:** 2026-10-28

**Authors:** Katharina Luttenberger, Carolin Donath, Lola Eversmann, Katharina Louisa Göttlicher, Julia-Sophia Scheuermann, Luisa Leonie Brokmeier, Anne Radegast, Pia Scheufele, Piotr Lewczuk, Vera Kiesel, Romi Kuntscher, Sebastian Meyer, Mark Hulm, Larissa Schwarzkopf, Anna Eichler, Oliver Kratz

**Affiliations:** 1 Section of Medical Psychology and Medical Sociology, Department of Psychiatry and Psychotherapy, Uniklinikum Erlangen, Friedrich-Alexander-Universität Erlangen-Nürnberg (FAU), Erlangen, Germany; 2 Department for Psychosomatic Medicine and Psychotherapy, University for Continuing Education (UWK), Krems, Austria; 3 Department of Child and Adolescent Mental Health, Uniklinikum Erlangen, Friedrich-Alexander-Universität Erlangen-Nürnberg (FAU), Erlangen, Germany; 4 Department of Psychiatry and Psychotherapy, Uniklinikum Erlangen, Friedrich-Alexander-Universität Erlangen-Nürnberg (FAU), Erlangen, Germany; 5 Department of Neurodegeneration Diagnostics, University Hospital Bialystok, Bialystok, Poland; 6 Institute of Medical Informatics, Biometry and Epidemiology (IMBE), Friedrich-Alexander-Universität Erlangen-Nürnberg (FAU), Erlangen, Germany; 7 Center for Mental Health and Addiction Research, Institut für Therapieforschung (IFT), Munich, Germany; 8 Institute for Medical Information Processing, Biometry, and Epidemiology (IBE), Ludwig-Maximilians-Universität München, Munich, Germany

**Keywords:** Depression, Bouldering, Rock climbing, Bouldering-Psychotherapy, Psychotherapy, Adolescents, Group therapy, Exercise, Randomized controlled trial

## Abstract

**Background:**

Currently, there are hardly any validated group therapy programs for depressed adolescents in Germany. A recent meta-analysis confirmed a moderate antidepressant effect of exercise in adolescents with depressive symptoms. In addition, four recent systematic reviews reported positive effects of exercise interventions on psychological well-being, mood, and quality of life in adolescents with depression or depressive symptoms. We developed a manualized behavioral-therapy-based group program involving Bouldering-Psychotherapy (BPT) for adolescents (BPT-J) based on the adult version of BPT, which has already demonstrated its efficacy. The aim of the study is to evaluate the efficacy of BPT-J.

**Methods:**

The study is designed as a randomized, controlled, prospective, longitudinal, single-blind trial with two arms (BPT-J intervention group [IG], waitlist-control group [CG]). The primary aim is to investigate the efficacy of the BPT-J group therapy for adolescents for treating depressiveness. Eligible participants are adolescents with subclinical and clinical depressive symptoms between 13 and 18 years of age. The adolescents in the CG are offered participation in an identical intervention after t1. The 10-week intervention is accompanied by four data collection points for all participants: participants and their legal guardians fill out online questionnaires at the start of therapy (t0), immediately after IG therapy (t1), after CG therapy (t2), and 12 months after finalizing the intervention (follow-up, t3). In addition, structured video interviews are conducted at t0 and t1.

**Discussion:**

BooSt trial addresses the limited availability of evidence-based group interventions for adolescents with depressive symptoms in routine care. If efficacy is confirmed, BPT-J may represent a scalable, manualized, and potentially well-accepted treatment approach that could complement existing psychotherapeutic services for adolescents. Given the increasing demand and limited access to psychotherapy, the intervention may contribute to improving accessibility of mental health care. The group-based format may additionally support cost-effective implementation, reduce waiting times, facilitate peer support, and help reduce stigmatization associated with mental health treatment. Findings from the BooSt trial may further inform dissemination and implementation strategies in child and adolescent mental health care.

**Trial registration:**

ISRCTN, ISRCTN79659949, prospectively registered 19/02/2025, DOI: https://doi.org/10.1186/ISRCTN79659949, URL: https://www.isrctn.com/ISRCTN79659949.

## Introduction

### Background and rationale {6a}

Recent data indicate that depression in adolescents in Germany has increased about 30% since 2018 and remains at an elevated level, particularly among girls. The prevalence of depression in adolescents is approximately 8% [1–3]. These national trends align with the World Health Organization’s assessment, which identifies depression as a leading contributor to disease burden and suicide in young people globally, underscoring its significance as a major public health concern in adolescence [4].

Children and adolescents seemed to have particularly strong reactions to the stress associated with the COVID-19 pandemic [5, 6]. Data collected during the pandemic as part of a nationwide, population-based study showed increases in psychological stress and feelings of depression and anxiety [7, 8]. The global guidelines on depression recommend a staged treatment approach, ranging from psychosocial interventions to psychotherapy and pharmacological treatment, either alone or in combination [9]. In contrast to the effectiveness of medication in adults, the effectiveness of anti-depressant drugs in adolescents is limited [10]. Additionally, data show that only around one fifth of children and young people with signs of mental health problems have had contact with specialized care providers at least once [11]. Possible reasons for the infrequent utilization of specialist support by children and adolescents affected by mental health symptoms or their families lie in the psychotherapeutic care gap and the lack of knowledge in the population about the type, content, accessibility, or financing options of support services. At the same time, scientists agree that early professional action (i.e., selective or indicated prevention or treatment) can reduce the current and long-term burden of psychological symptoms in children and adolescents, particularly with regard to depressive illnesses, as reviews have shown [12, 13]. Delayed intervention, especially in the highly dynamic and vulnerable stage of puberty, often leads to significant deterioration and chronification of mental illness. The associated loss of important formative experiences often cannot be remedied [14, 15]. Furthermore, a recent review showed that selective prevention measures for at-risk groups are more effective in terms of efficacy and costs than universal prevention programs aimed at all children and young people [16]. Regarding a long-term burden, reviews have found a close connection between psychological symptoms in childhood or adolescence and mental illness in adulthood [17, 18].

Due to the sharp rise in demand for therapeutic services for children and adolescents, there are calls for an urgent expansion of group therapy in these age groups. With regard to efficacy, there is generally no difference between individual and group cognitive behavioral therapy approaches [19], but group therapies have a number of advantages: They are more cost-effective, have reduced waiting times, and facilitate mutual support and feedback among participants. In addition, group processes may help counteract stigmatization [20].

At present, few validated group-based therapy programs exist for adolescents with depression in German-speaking countries. The two best-known group programs for the treatment of depression, MICHI [21] and “Coping with mood problems [Stimmungsprobleme bewältigen]” [22], were examined in an outpatient setting in studies with *n* = 15 [23] and *n* = 24 adolescents [24], each without a control group.

Several recent systematic reviews have reported beneficial effects of exercise interventions on psychological well-being, mood, and quality of life in adolescents with depression or depressive symptoms [25–28]. In line with these findings, individual studies have shown that sports-based interventions may reduce depressive symptoms with moderate effect sizes and potential long-term benefits [29, 30]. In the follow-up measurements of a randomized controlled study by Helgadóttir et al. [31], improvements in depressive symptoms were maintained for more than 12 months. Such improvements were found in particular for light and high-intensity training [31]. In addition, recent research findings indicate that the social component of sporting activities can be important in the treatment of depressive symptoms [32]. In the multimodal treatment of mental illnesses, regardless of age, exercise therapy makes a decisive contribution to activating and strengthening physical, psychological, and social resources; offers a variety of therapeutic approaches to the patient’s experience and behavior; and alleviates symptoms specific to the disorder [33]. As a positive side effect, it also counteracts the trend toward the deterioration of health behavior in adolescents identified in recent decades due to increased screen time and decreased exercise time [7]. Exercise is also recommended in the American and British guidelines for the treatment of mild depression in children and adolescents [34, 35].

As an answer to these challenges, we designed a psychotherapeutic group treatment for adolescents based on cognitive behavioral therapy that includes practical physical experiences on the bouldering wall.

Bouldering-Psychotherapy (BPT), which we developed, has already been shown to be efficacious for adults [36–38] and has the potential to effectively address both problem areas (psychotherapeutic care gap and lack of exercise) and to apply the associated recommendations in combination [39]. Additionally, it is designed to be applied both in early preclinical and clinical stages of depressive symptoms. Integrating bouldering into a psychotherapeutic setting offers several advantages. It may reduce stigmatization, promote physical activity and mindfulness, and create a complex range of interactions among participants and between individuals and the climbing wall. These situations can amplify diagnostically relevant behaviors and provide immediate, experiential material for therapeutic work. Novel and often unexpected functional coping strategies for situations previously perceived as difficult, shame-inducing, or frightening can be developed with therapeutic guidance and subsequently transferred to everyday life. Based on these considerations and in adaption of the Bouldering-Psychotherapy Manual for Adults (BPT), a manualized group intervention for adolescents (Bouldering-Psychotherapy for Adolescents [Jugendliche]; BPT-J) was developed. Following evidence of its efficacy in adults with depression and its non-inferiority to traditional cognitive behavioral group therapy, the present study aims to evaluate the efficacy of BPT-J in adolescents.

### Objectives {7}

The aim of the present study is to investigate the efficacy of group-based Bouldering-Psychotherapy for adolescents (BPT-J) in Bavaria, Germany, with regard to the reduction of preclinical or clinical depressive symptoms. For this purpose, the BPT-J intervention group (IG) is compared with a waitlist-control group (CG) in a randomized-controlled, yet naturalistic design. The research hypotheses are as follows:

I. Patients in the IG improve significantly (*p* < .05) more than patients in the CG in terms of the severity of depression symptoms.
II. This improvement is clinically relevant in terms of effect size (Cohen’s *d* ≥ 0.35 or *η*^*2*^ ≥ 0.035).
III. The proportion of patients with clinically relevant improvement is higher in the IG than in the CG. A number needed to treat (NNT) of ≤ 5 is considered sufficient [40].

The effects on secondary outcomes (e.g., depression diagnosis (categorical), quality of life, self-efficacy, suicidality, comorbid psychological symptoms) will be investigated on an exploratory level.

### Trial design {8}

**Fig. 1:**
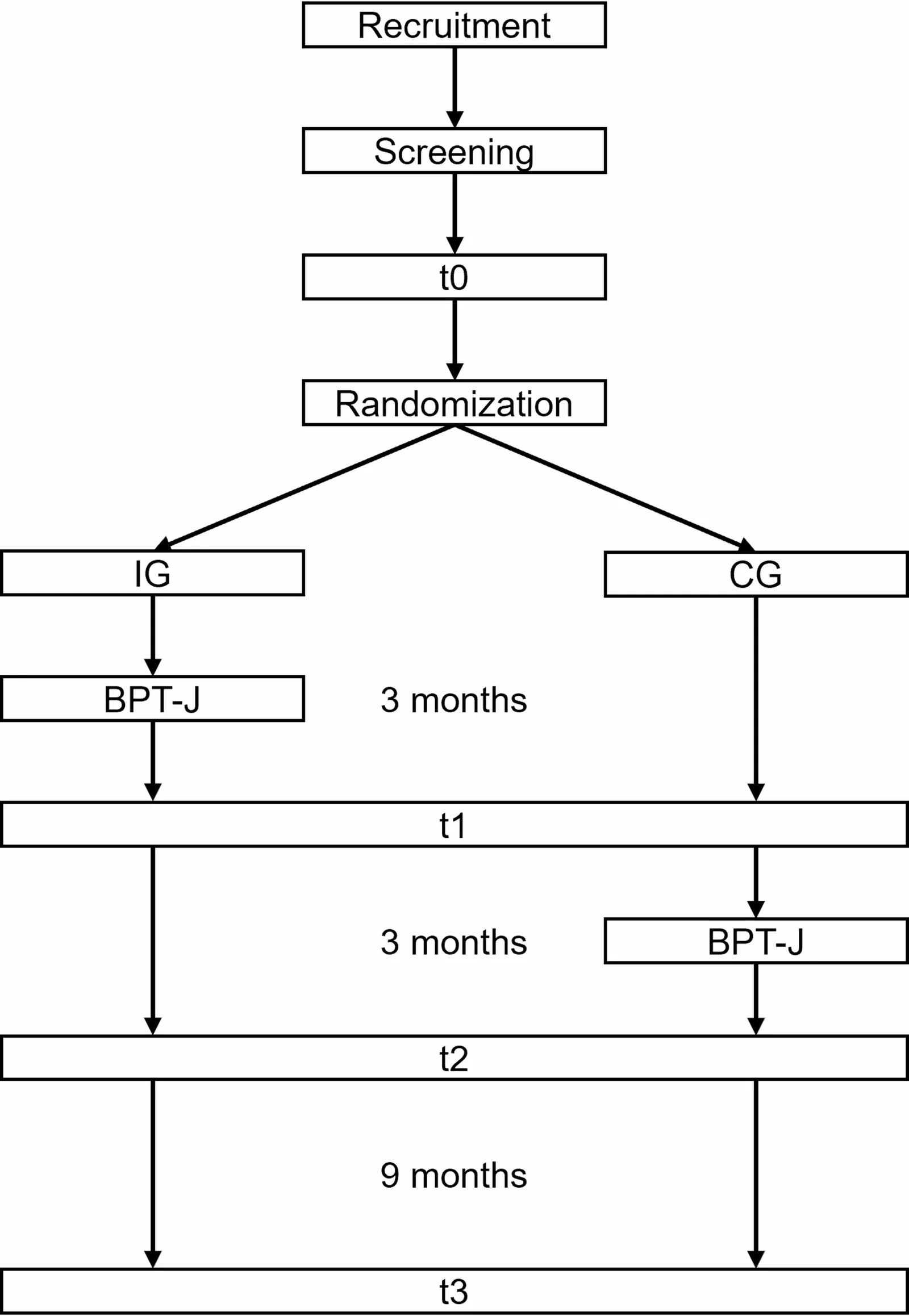
General course of the study for the IG and CG. *Note*. IG: intervention group; CG: control group; BPT-J: Bouldering-Psychotherapy for Adolescents

The study is designed as a randomized, waitlist-controlled, multi-center, interventional, prospective, longitudinal, single-blind study with two arms (IG: BPT-J group; CG: waitlist-control group). After completing a two-stage screening process and submitting a declaration of informed consent (from the participant and their legal guardian), participants are being randomly assigned to either a 10-week group intervention (IG) or a CG (see Fig. 1). For all participants, the trial is accompanied by four data collection points: All participants and their legal guardians are being asked to complete a series of online questionnaires at the start of IG therapy (t0), immediately after IG therapy (t1), after CG therapy (t2), and 12 months after t1 (follow-up, t3). In addition, structured video interviews are being conducted for categorical determination of the diagnosis of depression at t0 and t1. After the IG completes the BPT-J, the participants in the CG are being offered the opportunity to take part in an identical BPT-J. All participants who finish the 10-session BPT-J will be offered participation in an aftercare bouldering group.

**Fig. 2:**
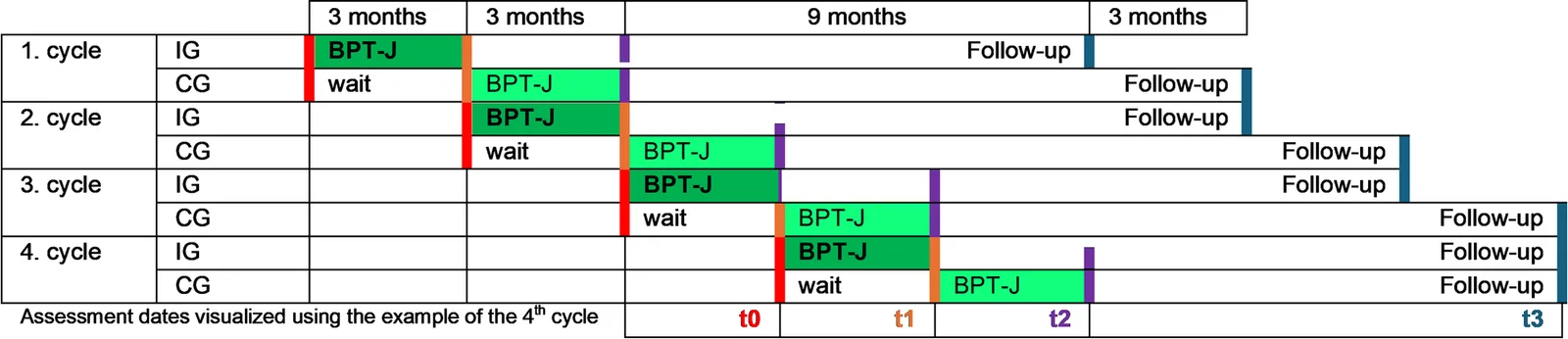
Visualization of the cycles for the IG and CG in one study center. *Note.* IG: intervention group; CG: control group; BPT-J: Bouldering-Psychotherapy for Adolescents

Four centers with at least four therapy cycles each are planned in the project. Figure 2 depicts one example center with the therapy cycles and data collection points.

## Methods: participants, interventions, and outcomes

### Study setting {9}

The study is being conducted with community-dwelling adolescents in four cities in Bavaria, Germany (Erlangen, Nuremberg, Bamberg, Regensburg). Adolescents can register online (www.boulderdichstark.de) for one of the cities. Therapy sessions are being delivered in cooperating bouldering gyms in each city for the IG and CG.

### Eligibility criteria {10}

In order to increase external validity, only a few inclusion and exclusion criteria are being applied. Inclusion criteria are acute depressive symptoms in at least a relevant subclinical range, operationalized by the Patient Health Questionnaire (PHQ-9) ≥ 6 [41], informed consent to participate in the study (especially with regard to randomized allocation and data collection) from both the participants and their legal guardians, the ability and time to participate in the intervention, and access to digital devices for video interviews.

Exclusion criteria are an age over 18 or under 13 years at t0, weight in an age percentile below 10%, or a body mass index (BMI) ≥ 35, taking medication for psychiatric symptoms, or a change in psychopharmacology medication ≤ 6 weeks before start of intervention. Further exclusion criteria are a planned inpatient stay (beyond a short-term crisis intervention) during the intervention period, physical contraindications to bouldering as judged by a medical doctor (physical complaints or pregnancy), diagnosed psychotic disorder or diagnosed manic illness, dependence on alcohol or illegal substances, and self-injurious behavior that goes beyond superficial scratching within the last 6 months. Self-injurious behavior without the intention to kill oneself is explored in a structured screening interview with items from the German version of the SITBI-G (Self-Injurious Thoughts and Behaviors Interview: German) [42, 43]. Individual outpatient psychotherapy is not a reason for exclusion.

For safety reasons, a step-by-step plan for dealing with suicidal thoughts and impulses is drawn up and confirmed by all participants during the screening. Interested individuals who are not willing or able to enter into such an agreement are not included. Instead, they receive a personalized consultation for suitable alternative offers.

**Fig. 3:**
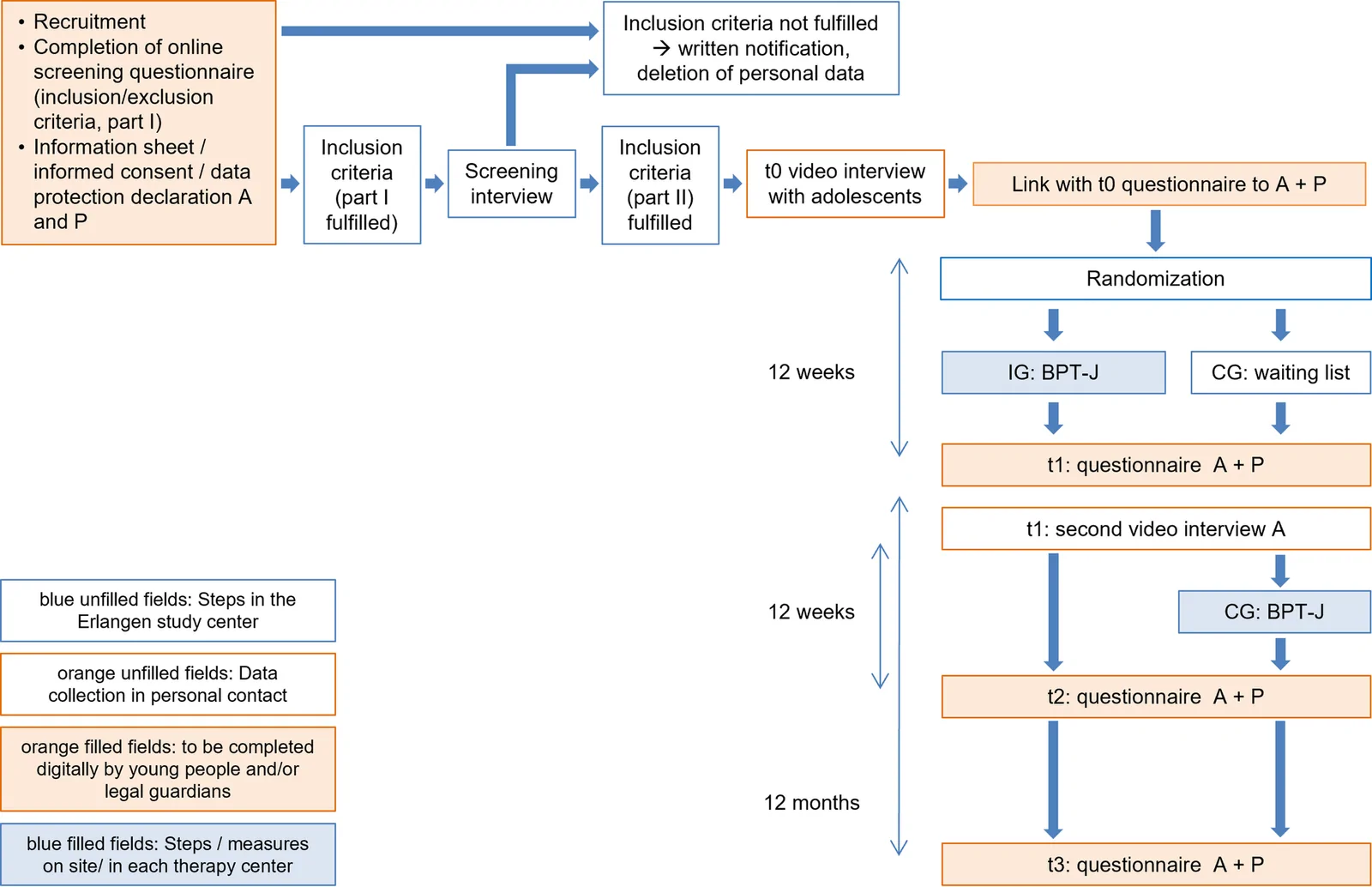
Flow diagram representing the screening and study procedure. *Note*. A: adolescent, BPT-J: Bouldering-Psychotherapy for Adolescents, P: parent/legal guardian

Screening takes place in a two-stepped procedure (Fig. 3): First, an online questionnaire is completed. If basic inclusion criteria such as age, weight, and depressive symptoms match, the remaining criteria are assessed in a structured screening interview. Subsequently, a definitive decision on study eligibility is made.

### Who will obtain informed consent? {26a}

All adolescents interested in participating and their legal guardians are invited to attend one of the general information sessions held by the study staff in each wave via Zoom for all four centers. Relevant information on the intervention and an explanation of the conditions of participation (e.g., randomization procedure) are being provided there. All questions are answered in detail at the event or in a one-on-one meeting. Furthermore, all information sheets (for adolescents and their legal guardians) as well as the data protection form are available on the website (www.boulderdichstark.de). Adolescents who decide to participate are asked to complete a screening questionnaire and screening interview on the inclusion and exclusion criteria. Written informed consent is obtained from the adolescents and at least one person with parental authority.

### Additional consent for the collection and use of participant data and biological specimens {26b}

Not applicable. No biological specimens are being collected.

### Interventions

#### Explanation for the choice of comparators {6b}

The study design incorporates a waitlist-control-group configuration, thereby enabling participants in the CG to participate in Bouldering-Psychotherapy as well. The implementation of a uniform intervention across all participants has been demonstrated to enhance adherence to the study protocol and mitigate the likelihood that the data will be missing at random. There is a high probability that the interventional BPT-J will also prove effective for adolescents because the BPT intervention has demonstrated effectiveness in adults. However, this study will be the first time that BPT-J has been tested in an adolescent population. A comparison with a waitlist-CG thus combines the advantages of ethical conduct (i.e., providing a potentially effective intervention to all participants) and of a first-trial intervention with treatment as usual (i.e., the freedom for all participants to attend whichever treatment is prescribed by their treating physician). Subsequent comparisons with other effective interventions might follow.

#### Intervention description {11a}

Participants are being randomized to one of two study arms.

##### Study arm 1: immediate start of IG BPT-J (Bouldering-Psychotherapy for Adolescents)

BPT-J is a manualized, body-oriented psychotherapeutic intervention that integrates bouldering with cognitive behavioral therapeutic techniques and a humanistic, client-centered framework. Bouldering is climbing without a rope on climbing walls at a height that permits the climber to jump off. The intervention consists of 10 consecutive group sessions. Each session lasts two hours and takes place weekly in the afternoon at a local bouldering gym. Each group comprises six to ten participants, who are guided by a team of two therapists. In each team, at least one of the therapists is a clinical psychologist or psychotherapist and at least one has additional training as a climbing instructor. All therapists have intensive experience working with groups of youth, have their own climbing experience, and have undergone two days of training specifically for the study. Regular supervision is being conducted (two visits to the group per wave, three supervision meetings per wave).

The curriculum, which has already been demonstrated to be effective in treating depression in adults, has been adapted and expanded in several feasibility groups to meet the needs and realities of young people’s lives (for BPT for adults, see: www.bouldering-psychotherapy.com). In BPT-J, participants are regarded as experts of their own lived experiences, and the definition of therapeutic goals lies with the adolescents themselves. Therapists support the articulation, clarification, and pursuit of these goals. The therapeutic rationale assumes that bouldering functions as a medium of transfer and amplification, whereby psychosocial challenges from everyday life become visible and experientially accessible through bouldering problems on the wall. In the protected therapeutic setting, participants are encouraged to experiment directly with new coping strategies and solution patterns, which are subsequently reflected upon and transferred to everyday contexts. Therapeutic themes are not introduced verbally but emerge from participants’ automated coping strategies during the bouldering exercises, with therapeutic work focusing on the individual themes that arise during each exercise. As a reflective and transfer-supporting element, BPT-J incorporates the use of a “resource carabiner.” Each participant is provided with a personal carabiner to which brief written notes capturing insights, positive experiences, or perceived strengths can be attached throughout the intervention. The resource carabiner serves as a concrete memory aid to support self-reflection and the transfer of therapeutic experiences to everyday life.

All 10 manualized sessions contain a fixed schedule and consist of three main components: an opening phase (approximately 15 min), including check-in and optional therapeutic input; an active bouldering phase (approximately 80 min), which begins with different exercises on group cohesion and a warm-up followed by specific bouldering exercises (see Table 1); and if any time remains, free bouldering, where all participants work on their individual “projects” with the support of the therapists. At the end of each session, there is a closing phase (approximately 25 min) for sharing experiences and for reflection and transfer. While the overall structure is identical across all sessions, therapists have flexibility in designing the opening and closing phases and the warm-up by drawing on a predefined pool of exercises. The bouldering phase is manualized, with exercises specified for each session. The opening and closing phases take place in a room that is separate from the bouldering gym (e.g., a yoga room) to ensure privacy and confidentiality.

**Table 1:** Overview of the bouldering-psychotherapy sessions

Session	Exercises
**1**	**Introduction to bouldering**
**2**	**Boulder transport**
**3**	**Kraken**
**4**	**Obstacle course**
**5**	**Route defining and elimination**
**6**	**Conductor and Siamese twins**
**7**	**Helping hands**
**8**	**Blind bouldering**
**9**	**Projecting**
**10**	**Resources and transfer to everyday life**

##### Study arm 2: Waitlist-CG

During the intervention phase, the CG is not receiving any study-specific additional therapy but has access to standard psychotherapeutic and psychiatric treatment options. Participants assigned to the CG are being offered exactly the same treatment rationale as the IG (participation in a 10-week BPT-J program in a group setting) after the conclusion of measurement points t0 (Baseline, pre-intervention) and t1 (after 3 months, post-intervention for the IG). Besides the wait time of approximately 3 months, the CG’s treatment plan is equivalent to the IG’s.

For all participants who complete their respective BPT-J group, an additional aftercare bouldering group is being offered on a weekly basis for at least 3 months.

### Criteria for discontinuing or modifying allocated interventions {11b}

Participation in the study as well as in the therapy groups is voluntary. The first of only two criteria that would lead to discontinuation of the intervention for a given participant, despite their own wishes, would be (a) worsening of suicidality to the degree that the agreement for dealing with suicidal thoughts and impulses leads to the next level, for example, in-patient care. In this case, therapists will initiate immediate help. The second criterion is (b) worsening of self-harming behavior or behavior endangering others, including serious violations of safety rules.

Under certain circumstances, participants can be allowed to join a group that is starting later, especially if they are members of the CG.

### Strategies to improve adherence to interventions {11c}

The therapists for each BPT-J group are independently documenting for each session if there were relevant deviations from the curriculum, if certain elements could not be delivered, or if there had been any adverse events. Furthermore, participant-specific content documentation is being used. Every team is being visited during each 10-session round at least once by a member of the study headquarters and given face-to-face feedback and supervision. Additionally, three supervision meetings are being held with one member of the study headquarters experienced in BPT and one licenced psychiatrist for children and adolescents. Participation in three supervisions per 10 therapy sessions is mandatory for each center (two for each therapist). Each adolescent’s participation is being closely monitored and documented. Participants with complete t1 data will be provided one free bouldering gym entrance for the aftercare group.

### Relevant concomitant care permitted or prohibited during the trial {11d}

While participating in the study, participants are permitted to take part in individual out-patient psychotherapy, to receive psychiatric medication if they had begun taking this medication more than 6 weeks before the study began, and to receive a change in the dose of an existing psychiatric medication. A prescription for a new anti-depressant treatment or in-patient stays are recorded and considered reasons for exclusion from the statistical analyses. However, such participants are allowed to remain in the group and attend the remaining sessions if possible.

### Provisions for post-trial care {30}

During the intervention, we are providing insurance that covers liability in case of death or gained, permanent disability caused to the patient by their participation in the study. As there is mandatory health insurance in Germany, and every study site has excellent medical treatment facilities, aftercare for injuries is secured for every participant at no personal costs. To sustain therapeutic gains and encourage ongoing physical activity, an open aftercare group will be offered from when the intervention ends until the last follow-up assessment. Participation in this group is voluntary and will be documented and factored into the follow-up evaluation.

### Outcomes {12}

#### Primary outcome

**Fig. 4:**
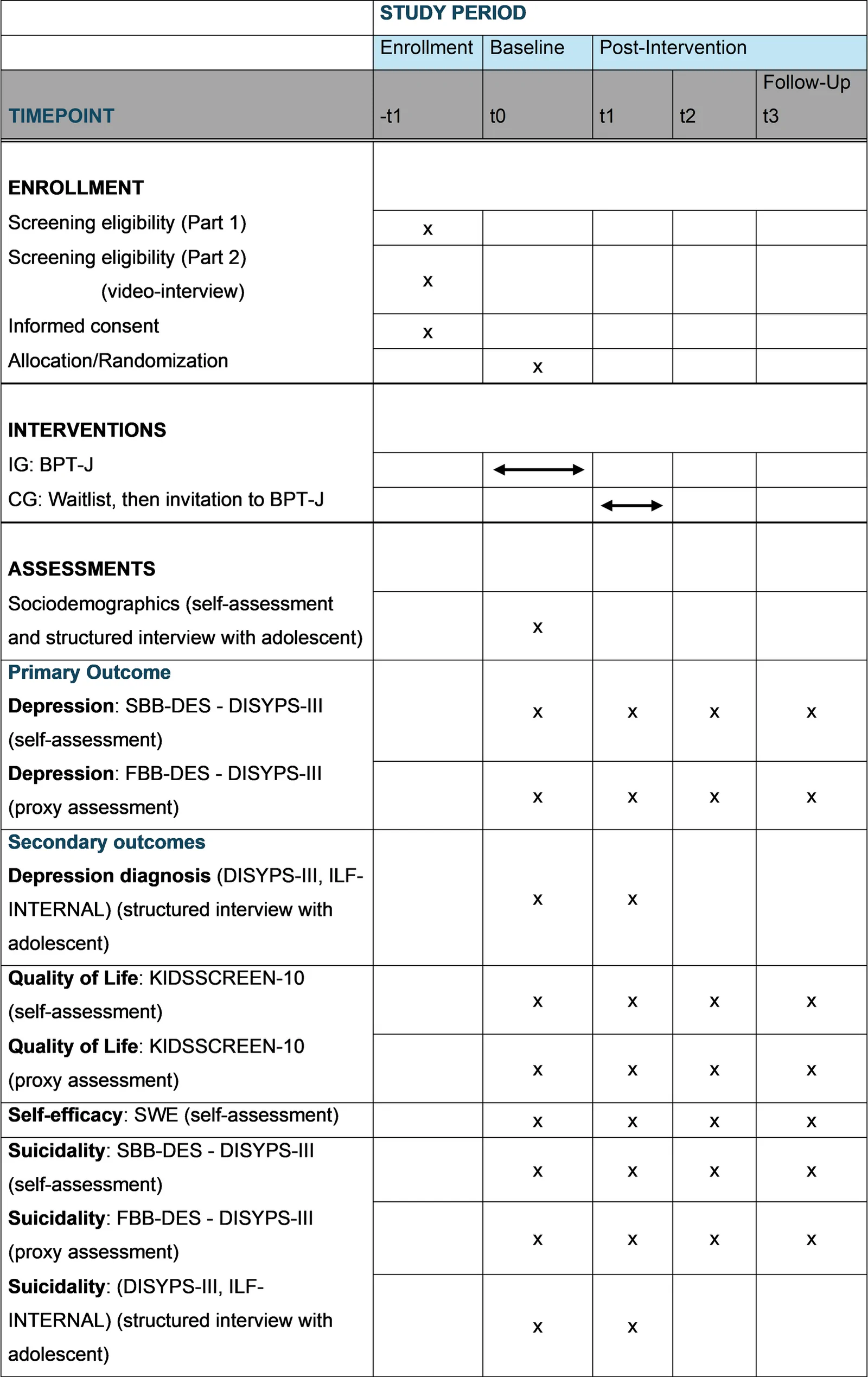
Overview of data collection instruments and measurement points. *Note*. IG = intervention group; CG = control group

The primary outcome is the severity of depression (dimensional) as measured by self- and proxy ratings. Therefore, the self- and parental/legal guardian questionnaires on depression (SBB-DES and FBB-DES) from the DISYPS-III (Diagnostic System for Mental Disorders as specified in the ICD-10 and DSM-V for Children and Adolescents - III) [44] are being used. The SBB-DES includes 29 items that capture the depressive symptom criteria as specified in the ICD-10 and DSM-5 and uses them to calculate a score. The response format ranges from 0 = *not applicable at all* to 3 = *particularly applicable*. The SBB-DES is designed for the age range of 11 to 18 years. Age-dependent norms are available for the SBB-DES, separately for boys and girls (stanine values). The FBB-DES assesses the depressive symptoms and the competencies of adolescents as proxy ratings (i.e., from the perspective of the parents/legal guardians). The FBB-DES items correspond to the SBB-DES items, including the response format. Corresponding age-differentiated norm values are available for the FBB-DES (stanine values). The SBB-DES and FBB-DES questionnaires are being administered at all four timepoints (t0 to t3; Fig. 4). The change from baseline to t1 (immediately after the intervention phase for the IG) in both the CG and IG will be compared as the primary analysis.

#### Secondary outcomes

##### Categorical diagnosis of depression

The (categorical) clinical diagnosis of depression assessed by trained raters in a structured clinical interview at t0 and t1 using the ILF-INTERNAL of the DISYPS-III is being measured to complement the dimensional assessment of depression [44]. The diagnostic interview includes an exploration of the symptom criteria, functional impairment, and level of suffering. Clinical standards are specified in the DISYPS-III manual for the procedure of categorizing the diagnosis on a 4-point scale (0 = *not present or age-appropriate*; 1 = *slightly pronounced*,* criterion not met*; 2 = *clearly pronounced*,* criterion met*; 3 = *very pronounced*). The frequency of all 4 categories will be analysed at t0 and t1.

##### Health-related quality of life

Adolescents’ health-related quality of life is being measured with the KIDSCREEN-10 [45] at t0, t1, t2 and t3 as a self-assessment rating and a proxy rating (legal guardians). It considers the physical, mental, and social aspects of well-being. The questionnaire contains 10 items, answered on a 5-point Likert scale. An overall mean value will be calculated, varying from 1 to 5, with higher values indicating a higher health-related quality of life. The change from t0 to t1 will be analyzed.

##### Self-efficacy

Participants’ self-efficacy is being measured with the scale for general self-efficacy expectations (SWE) [46] at t0, t1, t2 and t3 as a self-assessment. The questionnaire consists of 10 items, answered on a one-dimensional 4-point scale ranging from 1 (*does not apply*) to 4 (*applies exactly*). Subsequently, we will calculate a sum score that can range from 10 to 40, with higher values indicating greater self-efficacy [46, 47]. Numerous studies have demonstrated good psychometric parameters for the instrument; standardized values are available [48]. The change from t0 to t1 will be analyzed.

##### Suicidality

Suicidal behavior and suicidal thoughts are being recorded during the DISYPS interview at t0 and t1 and additionally assessed by self- and proxy ratings with two items (ranging from 0 to 3) from the DISYPS-III self- and peer-assessment questionnaires (SBB-DES and FBB-DES) at t0, t1, t2 and t3 [44]. The frequency of suicidality between t0 and t1 is compared.

##### Comorbidities

Other psychological comorbid symptoms are being recorded by the parents/legal guardians with the SDQ questionnaire (Strengths and Difficulties Questionnaire, German version) at t0, t1, t2 and t3 [49–51]. The instrument is used to quantify emotional and behavioral problems. The parents/legal guardians are asked to rate different behaviors concerning the last 6 months as *not applicable*, *partially applicable*, or *clearly applicable*. The questionnaire comprises 25 items, which are divided into five scales: emotional problems, behavioral problems, hyperactivity, behavioral problems with peers, and prosocial behavior. The first four scales are summed to form the total problem score. The change from t0 to t1 in that score will be analyzed.

#### Additional variables

##### Costs

To enable subsequent cost-effectiveness and cost-utility analyses from a societal perspective the participants’ all-cause utilization of health and supportive care services along with travelling distances (components of direct costs) as well as the participant’s and their legal guardians’ loss of working hours (indirect costs) will be assessed at t0, t1, t2 and t3 based on the previously applied FIM-K [52]. The FIM-K represents an adaptation of the established FIMA [53] to children and adolescents-specific cost components (e.g., parental accompaniment to inpatient treatment episodes, parental productivity). The eference period of assessment is set to the previous three month at each timepoint, to contextualize costs incurred during the 10-week intervention period appropriately. Reported health care utilization is subsequently monetized using Germany-specific unit costs [54], which are updated to the most recent base year (most likely 2025) via source research and inflation if required. Intervention costs (hall rental, manual, training of therapist team, hourly rates for BPT-J therapist team) are based on the study documentation.

##### QALYs gained

To reflect Quality Adjusted Life Years (QALYs) gained after the intervention ends, health-related quality of life will be assessed with the EQ-5D-Y via the adolescents’ self-ratings at each timepoint (see Fig. 4 and the “Health-related quality of life” section). The EQ-5D-Y is the child- and adolescent-specific version of the EQ-5D-3 L [55], which assesses problems in the five domains of mobility, independence, everyday activity, pain, and anxiety/depression with a 3-point scale (*no problems*, *some problems*, *major problems*). The individual answers can be converted into a person-specific health status (5³ = 125 possible statuses) for which a Germany-specific population-related tariff is available, enabling a transformation into QALYs [56].

##### Medication

Proxys are asked to report any medication use (prescription and “over-the-counter” pharmaceuticals) including trade name, pharmaceutical central number, package size, dosage (per day/week/month), route of administration and start date. In addition, the structured video interview specifically assesses initiation of antidepressant medication within the past six weeks.

##### Use of psychotherapy

The use of psychotherapy during the past three months is assessed using the FIM-K (see above), including the differentiation between individual and group therapy and the number of sessions. In addition, during the structured video interview, adolescents report lifetime and current psychotherapy, including onset and whether therapy is individual or group-based.

##### Adverse events

Adverse events (AEs) such as injuries, accidents, mental health crises, or hospitalizations and their intensity (mild, moderate, severe) are being assessed during the intervention period and during the year following the intervention. The intensity of these events is assessed on the basis of the extent of the impact on the participants’ lives and the resulting need for treatment. Mild AEs are defined as any injuries or emotional crises that can be handled without external care and have no lasting consequences (e.g., bruises, emotional outbursts handled within the therapy). Moderate AEs are defined as any injuries or emotional crises that need ambulatory external care but are reversible within weeks (e.g., sprains, emotional crises leading to professional contact with an external psychotherapist/psychiatrist or other health care provider). Severe adverse events (SAEs) would be any injuries or emotional crises that require hospitalization and have (potentially) irreversible consequences (e.g., head injuries, severe suicidal ideation, suicide attempts). Mild AEs are recorded by the therapists during the sessions (before t1 in the IG, before t2 in the CG). Moderate and severe events are being assessed in the following ways: IG/CG: by the therapists during the intervention; by the legal guardians in the questionnaires at t1, t2 and t3.

##### Use of sports

Current engagement in sports activities respectively exercise and if so – which kind and how many hours per week are assessed at all time-points. At baseline additionally, the engagement in sports/exercise before the start of the study is investigated.

##### Sports enjoyment

The enjoyment of sport is assessed by an adapted version of the Sports Enjoyment Scale for Adolescents (FEFS-J) which was specified for bouldering as sports activity. Two scales (“enjoyment” and “recovery”) with three items each are evaluated on a 4-point scale ranging from 1 (*never*) to 4 (*always*) at t1 (IG)/t2 (CG) (see Fig. 4).

##### Substance consumption

Life-time prevalence, 12-months-prevalence and age of first consumption concerning the substances alcohol, tobacco/e-cigarettes/vapes and cannabis are assessed. Items used were evaluated before and have been incorporated in representative epidemiological studies [57, 58].

### Participant timeline {13}

After completing a short digital initial screening form, which checks Part 1 of the inclusion and exclusion criteria (including depressive symptoms based on PHQ-9, age, height, and weight), participants are being automatically informed about whether they are suitable for detailed screening for study participation (see Fig. 3). Thereafter, at least within one month, interested and potentially eligible individuals participate in a diagnostic video screening interview to assess the remaining inclusion and exclusion criteria (screening Part 2) before being informed about whether they can participate in the study. The screening interview is followed by the structured clinical interview (DISYPS-III) for baseline assessment (t0) concerning clinical diagnosis of depression (categorical expression). Subsequently, self- and proxy-rated questionnaires are completed at baseline (t0) by the participants and their legal guardians if they are deemed eligible and give consent.

After participants complete the screening and baseline assessment (t0), the randomization, allocation, and start of the intervention for the IG takes place. Thus, the treatment phase for the IG is between t0 and t1, and the treatment phase for the CG is between t1 and t2 (see Fig. 4).

As soon as the participants of the IG complete the intervention, the depressive symptoms (categorical) are assessed again via video interview using the structured clinical interview (DISYPS-III) for all participants (IG and CG). Changes in participants’ current medication and/or eventually used parallel psychotherapeutic treatment are documented at t1. Furthermore, self- and proxy ratings made by participants and their legal guardians via questionnaires assessing the primary and secondary outcomes are collected at t1.

Questionnaires for assessing the primary and secondary outcomes by the participants and their legal guardians are also collected after the participants of the CG have completed their participation in the BPT-J (t2) and 12 months after each individual has completed their BPT-J participation (t3).

### Sample size {14}

The primary outcome of the study is the group difference in the pre- vs. post-treatment within-subject difference in depressive symptoms. Correspondingly, the sample size was determined from a two-sided t-test of the scores change between the two groups, acknowledging that more complex methods will also be used in the statistical analysis of the results.

We expect the standard deviation of the outcome variable to be twice as large at t1 compared with the standard deviation before the intervention. Since a meta-analysis of the effectiveness of group-based behavioral therapy in adolescents [48] showed only a small effect, we set the effect of the intervention of at least ¼ to ½ standard deviation of the difference in the outcome measured pre- vs. post-treatment. With this assumption and targeting a statistical power of at least 80% and a significance level of 5%, the study requires 97 to 127 participants per group, so between 194 and 250 in total.

In our experience, around 20% of those who apply to take part in a study do not meet the inclusion criteria. Thus, around 300 individuals have to be screened.

### Recruitment {15}

Participants are being recruited through a multi-faceted strategy aimed at maximizing sample representativeness. The initial outreach is targeting schools, parent associations, school psychologists, school district administrations, and community departments for youth and family services, with requests to broadly disseminate information about the study.

Additional recruitment efforts are including the distribution of informational materials (e.g., flyers, posters) in psychiatric clinics, psychotherapy practices, general practitioners and pediatric offices, pharmacies, and other psychological and social service institutions such as self-help groups and counseling centers as well as in bouldering gyms. Furthermore, psychotherapists, psychiatrists, and general practitioners in outpatient clinics, training outpatient centers, and private practices are being contacted and asked to share the study invitation with patients on their waiting lists.

To enhance visibility and accessibility, a dedicated project website was launched, and social media platforms (e.g., Instagram, TikTok) are utilized, including those affiliated with the University Hospital Erlangen and various local climbing gyms.

People interested in participating can use the screening links on the web page and directly begin enrolling in the study. Those agreeing to be contacted for the upcoming or a later cycle of the study are being contacted by e-mail. Before each wave, press releases are being issued and addressed to various local newspapers. For recruitment and informed consent, several public project information sessions are being organized online for each cycle. To reach a maximum number of adolescents, recruitment and therapy periods are scheduled for outside the Bavarian school holidays.

### Assignment of interventions: allocation

#### Sequence generation {16a}

A stochastic minimization algorithm is being employed to reduce group imbalances. It includes the following stratification variables: age, sex, and severity of depressive symptoms [59]. More specifically, 1,000 random permutations of group allocations with equally sized groups are being generated for the new participants in each block.

The first permutation that minimizes the summed chi-squared statistics for group differences regarding age group (< 16 vs. ≥16), sex, and symptom severity (0–3) is selected. The resulting allocation is not predictable. The randomization algorithm is implemented in the statistical software environment R [60].

#### Concealment mechanism {16b}

We are assessing the pretest data before randomization. Every participant is given a pseudonym, which is sent to the statistician with only the information that is relevant for stratification. After gathering the pretest data for all participants for the respective wave, the statistician performs the randomization and provides an allocation list to the study center. All participants and their parents are then informed about when and where they should meet for the first session.

#### Implementation {16c}

The blockwise randomization per study cycle and center is being carried out externally by the Institute for Medical Informatics, Biometry and Epidemiology (IMBE) of the Friedrich-Alexander-University Erlangen-Nürnberg. The statistician transmits the group assignments back to the study center. After randomization, the participants are informed about their allocation (immediate group start: IG, delayed group start: CG) and are given detailed information about their group participation by the study staff.

### Assignment of interventions: blinding

#### Who will be blinded {17a}

All individuals who are collecting data, particularly the interviewers, are blinded to participants’ group assignments. At the beginning of each interview, participants are reminded that their allocation is confidential and that they must not tell the interviewer which group they are in. Due to the nature of the intervention, neither therapists nor participants can be blinded. However, the therapists do not know which participants have been allocated to the IG or CG except for those in the first (only IG) and last (only CG) intervention cycles.

#### Procedure for unblinding if needed {17b}

Interviewers should not be unblinded until after the last interview. Any questions that arise about therapy are managed by the study center in direct contact with parents, participants, or therapists.

### Data collection and management

#### Plans for assessment and collection of outcomes {18a}

The main outcome data are being assessed via self-report questionnaires from the respective adolescent and by proxy ratings from their parents, as well as via clinical interviews. This approach allows for crosschecks regarding potential issues with reliability or validity. To test interrater reliability, 10% of all video interviews are being assessed by two differently paired raters. The quality of the data is being ensured by strict data monitoring at the study center across the entire study period. For an overview of the data collection instruments used, see the Outcomes section and Fig. 4.

#### Plans to promote participant retention and complete follow-up {18b}

All outcome data are being collected for all participants even if the respective participant discontinued the intervention (for the list of outcomes, see Fig. 4).

To retain participants in the trial and promote their participation in the interviews and surveys, we implemented a number of incentives for participants: Participants from both groups are being given a voucher for a free session at their local bouldering gym (about 12 €) after completing t1 and another voucher after completing t3. Participants are only allowed to take part in one of the IG if they participated at all timepoints before their respective intervention. After the 10 weeks of the intervention, all participants with complete data are being offered participation in an aftercare bouldering group.

#### Data management {19}

The interviewers have been thoroughly trained and are being supervised by the staff at the study center. Before each timepoint, all interviewers receive special training and practice under supervision. Weekly supervision sessions are held by members of the scientific staff. Data are recorded via proven online tools (RedCap) [61] and exported on a regular basis. Allowed data ranges and data dropdown lists have been programmed into the software to reduce the likelihood of incorrect data entries. Every participant is given his or her own unique data identifier, which is included in RedCap and double-checked at the beginning of each interview. Questionnaires are programmed with personalized links and automatically sent by the RedCap software.

#### Confidentiality {27}

The data are being collected via interviews or online questionnaires. Additionally, therapists are collecting data on the intervention sessions (adherence, participation, and adverse events). Data collection is being conducted with the data-secure RedCap participant data collection system, which is hosted on the University Hospital Erlangen’s server. The interviews are being conducted with the Samedi software, certified by the National Association of Statutory Health Insurance Physicians (KBV – Kassenärztliche Bundesvereinigung).

Data handling is in accordance with European and German data protection laws, and the information sheet contains a data protection declaration. No personal data are being stored in the data collection system, except for participants’ e-mail address, for which consent has been given. Other sensitive data collected during the video interviews or in the questionnaires are being pseudonymized to guarantee the ability to match the data across the different measurement points and store that data in password-protected devices and programs. The names of the participants, their contact information, and the corresponding codes are being recorded in a separate, password-protected document. The key file for pseudonyms and participants’ data (names and addresses) is being stored securely on the University Hospital Erlangen’s server and is password-protected. Only members of the study team have access to this list. The informed consent sheets are being digitalized and stored in password-protected devices. The published material will not contain any identifying information.

#### Plans for collection, laboratory evaluation, and storage of biological specimens for genetic or molecular analysis in this trial/future use {33}

Not applicable. No genetic or molecular data will be collected or analyzed.

### Statistical methods

#### Statistical methods for primary and secondary outcomes {20a}

##### Hypothesis I

The Primary Outcomes (severity of depression, self- and proxy assessment) will be expressed as a linear combination of 29 ordinal random variables (answers to the questionnaires) per participant, whereas particular distributions of those variables (i.e., responses to the questionnaire items) across the participants are not relevant in this study and the importance of all questionnaire items for the assessment of the severity of depression are equally weighted. Under the assumption that these underlying variables are independent and conditional on a participant (which we acknowledge is not completely true), we can take advantage of the Central Limit Theorem (CLT) and treat the resulting average across the 29 responses as approximately normally distributed. The design of the study—with the outcome variable (and the explanatory variables) collected at baseline and then at three follow-up timepoints—leads to a hierarchical structure of the data with occasions (Level 1) nested within participants (Level 2 clusters). Therefore, the data will be analyzed with Linear Mixed Models (LMM). In the main (fixed effects) structure, the treatment effect will be represented by the interaction between the group and the occasion, possibly in presence of covariates. Since it is rather unlikely that the evolution of the outcomes will be linear in time, the occasions will be treated as a categorical variable. In the random part, intra-subject homogeneity will be addressed by including a subject-specific random intercept, and the conditional (on participants) dependence of the residuals across occasions will be reflected by the appropriate structure of the residual covariance matrix (e.g. 1st order autoregressive). In addition, a hybrid model will be formulated in which a random intercept (hierarchical) LMM formulation will be combined with a marginal formulation, with exponential structure of the residuals to reflect serial correlation of the outcomes that do not approach zero and that are collected at unequally spaced timepoints [62]. The main structures of the models will be modified and tested for potentially better fit by adding/removing covariates in a forward-backward non-automatic strategy. Likelihood Ratio Tests and/or Information Criteria (in case the candidate models are not nested) will be used. Model parameters will be estimated by maximization of the likelihood (MLE). Modeling will be performed in Stata (StataCorp, College Station, TX, USA). To test the hypotheses, *p* < .05 will be considered significant.

##### Hypothesis II

In addition to the testing of Hypothesis I, effect sizes will be calculated both for the group difference and for improvement during the therapy period. The secondary outcome variables (depression diagnosis (categorical), quality of life, self-efficacy, suicidality, comorbid psychological symptoms) will be tested in an exploratory fashion using Generalized Linear Models (GLM) and respectively LMM.

##### Hypothesis III

The clinical relevance of the change in symptoms in Hypothesis III will be tested analogously to Goletz et al. [63] and Walter et al. [64], on the basis of both external assessment and self-assessment using two criteria.

1. Criterion 1 was defined as dichotomized depressive symptoms based on age-normed stanine values. Stanine values ≥ 7, indicating above-average symptom severity relative to the normative sample, were classified as *conspicuous*, whereas stanine values < 7 were considered *inconspicuous*.
2. Additionally, the RCI (Reliable Change Index) will be used to determine the course of symptom severity between t0 and t1. The RCI is calculated according to Jacobson et al. [65] and based on Hanisch et al. [66].

Using the combination of both clinical relevance criteria, the participants will be classified into five groups after completion (see Table 2), and relative frequencies will be determined. The proportion of adolescents with clinically relevant improvement in their depression scores (response) and the proportion of adolescents who improved into the normal range (remission) will be compared in the response analysis with the adolescents whose depression scores did not improve. For this purpose, a regression model for binomial outcome will be calculated with the variable response/remission as the dependent variable (DV) and the factors of influence discussed in the literature as independent variables (Hypothesis III). Additionally, the number needed to treat will be calculated.

**Table 2:** Combination of criteria for assessing the clinical relevance of changes in symptoms

	*Improved according to RCI (> 1.96)*	*No change according To RCI (–1.96 < X < 1.96)*	*Deterioration according to RCI (< − 1.96)*
***Test value inconspicuous after dichotomous classification (Cut-Off)***	Remission (response)	Subclinical but stable (non-response)	Deterioration (non-response)
***Test value conspicuous after dichotomous classification (Cut-Off)***	Improvement (response)	No change and clinically relevant symptoms (non-response)	

#### Interim analyses {21b}

There will be no interim analyses of depression scores. As a result of repeated tests of the same hypotheses, there is an increased risk of making incorrect conclusions about the efficacy and safety of an intervention [67]. For unintended adverse events, see {22}.

#### Methods for additional analyses (e.g., subgroup analyses) {20b}

##### Cost-effectiveness

The cost-effectiveness of BPT-J will be assessed immediately after the intervention (main analyses), from the perspective of a healthcare payer (primary analyses), and from a societal perspective (secondary analyses). From the payer’s perspective, only direct costs borne by the statutory health insurance will be considered, whereas from the societal perspective, out-of-pocket payments, informal care, and indirect costs will also be accounted for. Costs will be modeled along a gamma distribution with a log-link in order to take appropriate account of the right-skewed distribution [68]. Here, depending on the proportion of participants with zero costs, either one-part gamma models or two-part gamma models will be applied [69]. The effect side is reflected by the primary clinical outcome (FBB-DES, SBB-DES) and within a sensitivity analysis by QALYs gained, assuming a zero-truncated Gaussian distribution for both parameters. For the primary ITT analysis, missing data immediately after the intervention will be imputed with multiple imputation. The analysis model is structured likewise as the main model for the primary outcome(s).

Subsequently, incremental cost-effectiveness ratios (ICER), which reflect the adjusted mean difference in total costs divided by the adjusted mean difference of effects (FBB-DES, SBB-DES, QALYs gained) will be calculated. To align the cost-effectiveness analysis with the analysis of clinical effectiveness, the same covariate structure will be applied. To address uncertainty around the point estimate of the ICER, 1,000 simultaneous bootstrap replications will be performed and will be translated into cost-effectiveness planes. In addition, cost-effectiveness acceptance curves (CEAC) will be created to determine the probability of cost-effectiveness for different willingness-to-pay thresholds [70, 71]. To check the robustness of the results, the cost-effectiveness analyses will be repeated in the per protocol sample.

#### Methods for analyses to handle protocol non-adherence and any statistical methods to handle missing data {20c}

Access to bouldering groups is being given only at the respective timepoint on the basis of the randomization. Therefore, no member of the CG can participate in the IG. Participants who are randomized into the IG but do not show up to the bouldering sessions are analyzed as members of the IG. “Dropout” can be included as a covariate if necessary.

As stated in the previous section {20a}, the data will be analyzed with LMM by maximization of the likelihood functions. In contrast to the somewhat old-fashioned ANOVA family, estimates from regression models—as long as they rely on the likelihood theory—are asymptotically consistent in the presence of missingness, assuming that the data are Missing At Random (MAR) [62]. This consistency is due to the property of the likelihood estimation called ignorability, which holds true if scientific interest is not directed toward the missingness mechanism and the parameter spaces of the outcomes (fixed effects and covariance parameters), and the missingness processes are disjoint [72].

#### Plans to give access to the full protocol, participant-level data, and statistical code {31c}

Pseudonymized data without personal information potentially identifying patients will be made publicly available via an open data repository after all analyses have been completed.

### Oversight and monitoring

#### Composition of the coordinating center and trial steering committee {5d}

The trial coordination is being led by two psychologists, one a Professor of Psychotherapy Research and fully approved CBT-psychotherapist and the other a Professor of Medical Psychology and Sociology. Organization of the day-to-day running of the trial and data management is being coordinated by two M.Sc. Psychologists. Psychotherapists working on the trial are further supervised by a chief physician in Child and Adolescent Psychiatry. An additional M.Sc. Psychologist is supporting the organization and overseeing data management.

#### Composition of the data monitoring committee, its role, and reporting structure {21a}

The data monitoring committee (DMC) consists of one full professor of clinical psychology and psychotherapy for children and adolescents, one full professor from the institute of biometrics and epidemiology, and one senior physician in the clinic for psychiatry and psychotherapy. The DMC is completely independent from the sponsor and has no competing interests. The DMC is regularly informed about the progress of the study and consulted before all important milestones. In particular, the DMC monitors the occurrence of adverse events and decides whether the termination criteria have been met.

#### Adverse event reporting and harm {22}

Adverse events (AEs) are categorized according to severity into mild, moderate, and severe adverse events. Severe adverse events (SAEs) require long-term care and include, for example, severe head injuries, spinal cord injuries, mental health crises leading to hospitalization, suicide attempts, and death. Moderately severe events require treatment but are temporary, such as injuries to bones in the extremities or torn/strained ligaments. Minor adverse events are temporary injuries that do not require treatment, such as abrasions or bruises. AEs are documented weekly by the therapists during the intervention periods. In addition, moderate and severe adverse events are assessed at all post-intervention timepoints in the proxy ratings of parents or legal guardians.

After each timepoint, the frequency of AEs in the IG and CG is compared and presented to the DMC. The final decision about the termination of the intervention lies with the DMC. In the case of moderate AEs, the intervention will be terminated if there are three more AEs in the IG than in the CG if the AEs are determined to be a direct result of the intervention.

Mild AEs will be recorded but will not result in the termination of the program. If an SAE occurs in the bouldering group and is determined to be a direct result of the intervention, the program will be terminated if the intervention is determined to be directly responsible for the SAE.

As in all physical activities, there is a risk of injury associated with participation in the BPT-J. In a study on sport climbing injuries, an average of 0.2 injuries per 1,000 h of climbing (which carries a higher risk of serious injury than bouldering) was found in a sample of approximately 2,000 climbers [73]. In our study, bouldering time is limited to a maximum of 10*2 hours per patient. Serious injuries are therefore not to be expected. In our previous studies, no SAEs occurred in more than 4,000 h of bouldering. All therapists are thoroughly trained by a bouldering instructor from the German Alpine Association, and special safety plans (no jumping, strict warm-up routine) are implemented.

#### Frequency and plans for auditing trial conduct {23}

Due to the low-risk nature of the intervention, no formal trial auditing has been established. Instead, attendance and therapy progress as well as AEs in the IG will be documented weekly by the therapists. This documentation will be cross-checked by the coordinating center. The overall conducting of the trial is being monitored through weekly meetings of the coordinating center to review the progress of the study, ensure adherence to the trial protocol, and address challenges. Data integrity, assessment, and documentation are being overseen by the DMC (see {21a}).

#### Plans for communicating important protocol amendments to relevant parties (e.g., trial participants, ethical committees) {25}

The external funder, the Bavarian State Ministry of Health, Care and Prevention, is continuously being informed about the progress of the study. If any important protocol modifications need to be made, we will inform the Ethics Committee, the funder, and the trial registry platform ISRCTN.

### Dissemination plans {31a}

The research group intends to publish the data generated from this study in peer-reviewed journals. In addition, results will be communicated at international conferences, national conventions with the funders, the study’s webpage, and the press.

## Discussion

The study is designed as a naturalistic randomized waitlist-controlled trial with high external validity to provide evidence on the efficacy and clinical relevance of short-term BPT-J for the treatment of (subclinical) depression.

In recent years, numerous epidemiological studies have shown a growing psychological burden among children and adolescents. This trend intensified during the COVID-19 pandemic and could not be reversed even after restrictions had been lifted. For years, suicide has been the leading cause of death among adolescents between the ages of 10 and 24 [74] in Western societies—surpassing traffic accidents, violence, and medical conditions. Many suicides are preceded by a mental disorder, most commonly depression.

In a recent study, 18.8% of adolescents in the 9th -12th grades reported regularly thinking about their own death, and 8.9% had already attempted suicide in the past [75]. The everyday lives of many adolescents have increasingly shifted into the digital sphere, and the average screen time among adolescents in Germany is 201 min per day [76]. Besides the often-discussed potential negative impacts on mental health, excessive screen time also reduces physical activity and thereby indirectly increases the risk of developing psychological disorders. Evidence from longitudinal observation suggests that replacing screen time with physical activity has mental health benefits for adolescents [77].

At the same time, many countries, including Germany, currently face an inadequate psychiatric and psychotherapeutic care infrastructure, with waiting times for outpatient therapy of up to 12 months. Inpatient facilities are also experiencing capacity shortages and are often limited to acute crisis interventions. In research as well, psychotherapy for adolescents is not as well-established as research for adult patients: Compared with the vast number of psychotherapy studies conducted with adults (see recent meta-analyses), psychotherapy research with high scientific standards involving depressed adolescents is still scarce [78]. Moreover, studies have consistently found smaller effect sizes for psychotherapy in adolescents compared with adults [79, 80].

The approach presented in this paper—a sport-based group psychotherapy program—aims to address both the psychological and the physical components. As a cognitive behavioral group therapy, it translates effective therapeutic mechanisms such as emotional exposure, practicing alternative behaviors, and training social skills into the experiential context of bouldering. Physical activity shifts the attentional focus from external appearance (“How do I look?”) to internal experience (“How does this movement feel?”), allowing the body to be experienced as a source of self-efficacy.

The typical age-appropriate shyness and fear of discussing emotional problems in a group setting are mitigated by the action-oriented nature of Bouldering-Psychotherapy: Work focuses on what emerges in the moment. Dysfunctional strategies are addressed through brief one-on-one interventions on the wall rather than in front of the entire group.

At the same time, the practical consequences of adopting more functional strategies can be experienced immediately and can be physically anchored. Furthermore, Bouldering-Psychotherapy, as a group-based intervention, is economically efficient and suitable for providing treatment to a larger number of adolescents in the healthcare system. The well-established program for adult patients with depression [36, 37] was modified according to the needs and lived experiences of adolescents in three feasibility groups with adolescents.

The study is designed as a naturalistic randomized waitlist-controlled trial. Only clinically relevant exclusion criteria are applied, providing high external validity. With up to 250 adolescents, the sample size will be large enough to detect moderate effect sizes and is larger than in most adolescent outpatient trials [81].

Suicidality is higher among adolescents with mental illness [82] and is therefore being strictly monitored throughout the study. In the therapy sessions, therapists are following good clinical practice in monitoring suicide risk. In case of acute risks, suicide risk management plans have been established. Therapists are receiving regular supervision by psychotherapists and psychiatrics who specialize in adolescent psychotherapy. While the primary outcome is assessed via self-report and proxy ratings, the assessment of the categorial outcome, which serves as basis for the analysis of clinical relevance, is assessed by blinded raters.

As the analyses also have a strong focus on clinical relevance, the study is designed to be useful for clinicians as well as for researchers. Including participants who already show subclinical symptoms may give important insights into the efficacy of early interventions.

This study also comes with a handful of limitations:

Including participants with subclinical depression reduces the potential span of improvement, thereby leading to a reduction in the achievable effect size. This limitation might be a threat to detecting significance.

During and after the intervention, participants are free to start or continue other individual therapeutic treatments without any disadvantages. Already ongoing treatment plans are not changed by their participation in this study. Therefore, possible confounding effects due to simultaneous interventions cannot be ruled out. However, ongoing and new treatments as well as changes to treatment plans during the study are recorded in the video interviews, and such data will be taken into account in the analyses. For ethical reasons and to increase therapy adherence, all adolescents are being offered the same intervention at different timepoints. Thus, the main outcome is the comparison between the IG and CG at post-test. Follow-up analyses will provide data on the course of symptom severity and can no longer be seen as a controlled comparison.

## Conclusion

This study is distinguished by its naturalistic design, resulting in high external validity, and a long follow-up period of 12 months. We are applying high research standards by having the interviews conducted by blinded interviewers and by meeting a possible selection bias with strict randomization of participants. To our knowledge, there are very few studies with out-patient adolescents with depression with a comparable quality and sample size.

## Trial status

Protocol version 1.0, 05/2026. The overall start date of the study was October 1st, 2024. Recruitment began on May 6th, 2025 and will continue until September 5th, 2026.

## Data Availability

No datasets were generated or analysed during the current study.

## Abbreviations

AE: Adverse events
BMI: Body mass index
BPT-J: Bouldering-Psychotherapy for Adolescents
CG: Control group
IG: Intervention group
NNT: Number needed to treat
PHQ-9: Patient Health Questionnaire
SAE: Severe adverse events
SDQ-Deu: Strengths and Difficulties Questionnaire – German Version
SITBI-G: Self-Injurious Thoughts and Behaviors Interview – German Version
SWE: Self-efficacy expectation
